# The composite microbial agent controls tomato bacterial wilt by colonizing the root surface and regulating the rhizosphere soil microbial community

**DOI:** 10.3389/fmicb.2025.1559380

**Published:** 2025-04-30

**Authors:** Shuangxi Zhu, Xiaojian Chang, Nana Liu, Yanhui He, Jianwen Wang, Zhansheng Wu

**Affiliations:** ^1^Xi’an Key Laboratory of Textile Chemical Engineering Auxiliaries, Key Laboratory of Textile Dyeing Wastewater Treatment Universities of Shaanxi Province, School of Environmental and Chemical Engineering, Engineering Research Center of Biological Resources Development and Pollution Control Universities of Shaanxi Province, Xi’an Polytechnic University, Xi’an, China; ^2^Agricultural Technology Extension Center of Xi’an, Xi’an, China

**Keywords:** tomato seedlings, bacterial wilt, composite microbial agent, soil microbial community, root exudates

## Abstract

**Introduction:**

Bacterial wilt caused by *Ralstonia solanacearum* seriously affects the healthy growth of tomato seedlings. Biocontrol microbes have been used to manage tomato bacterial wilt. Herein, we aim to investigate the behavior of the *Enterobacter hormaechei* Rs-5 and *Bacillus subtilis* SL-44 composite microbial agent (EB) in the rhizosphere soil, and assess its impact on both the soil microbial community and tomato plant growth in this study.

**Methods:**

The plate confrontation experiment and the pot experiment were respectively used to explore the control ability of EB against *Ralstonia solanacearum* and bacterial wilt disease. The absolute quantitative PCR (AQ-PCR) was employed to investigate the migration ability of EB in the rhizosphere of tomatoes, and the chemotactic response of EB to tomato root exudates was analyzed by the swimming plate method. Scanning electron microscopy was utilized to study the biofilm formation of EB during its colonization on the root surface of tomatoes. Finally, high-throughput sequencing was adopted to analyze the impact of EB on the microbial community in the rhizosphere soil of tomatoes after being infected by *Ralstonia solanacearum*.

**Results:**

The absolute quantitative PCR and scanning electron microscope showed that the EB could migrate and efficiently colonize the elongation zone of tomato roots to form a biofilm. In addition, the EB exhibits a chemotactic response to tomato root exudates like sucrose, leucine, glutamic acid, and aspartic acid. The pot experiment demonstrated that the EB can reduce the incidence of tomato bacterial wilt from 77.78% to 22.22%, and significantly increase the biomass, physicochemical properties, and rhizosphere soil nutrient contents of tomato seedlings. Besides, the relative abundance of beneficial bacteria such as *Massilia*, *Pseudomonas*, *Bacillus,* and *Enterobacter* increased, and the fungi community diversity was improved.

**Conclusion:**

Overall, the EB can reduce the amount of *Ralstonia solanacearum* in rhizosphere soil, and then control tomato bacterial wilt directly. Besides, the EB can migrate to the root under the induction of tomato root exudates and colonize on the root surface efficiently, thereby indirectly regulating the soil microbial community structure and controlling tomato bacterial wilt.

## 1 Introduction

The bacterial pathogen *Ralstonia solanacearum* can invade from the wounds at the root or stem base of the host, and survive in the soil for a long time, which causes the stems and leaves to wilt without a normal supply of water (Su et al., 2022a). The tomato is a vital vegetable crop globally, with an annual global production of about 160 million tons, and assumes a pivotal role in human dietary patterns. The tomato bacterial wilt caused by *R. solanacearum* severely impacts the growth of plants, often resulting in extensive wilting and mortality, leading to significant losses of 35%–90% of tomato yield (Su et al., 2022b). The bacterial wilt, a highly destructive soil-borne disease, represents one of the most extensively prevalent and detrimental plant diseases worldwide (Prokchorchik et al., 2020).

*Ralstonia solanacearum* exhibits a broad range of hosts and distribution, possesses a complex pathogenic mechanism, and demonstrates strong survival and transmission capabilities (Prior et al., 2016). Therefore, there are no effective methods for completely controlling tomato bacterial wilt (Md Meftaul et al., 2020). Thus, prevention is the main method to prevent tomato bacterial wilt from invading and spreading to plants. At present, many techniques have been used to control tomato bacterial wilt, with pesticides being the most widely used (Qi et al., 2022). However, pesticides can cause environmental pollution and food safety in preventing and controlling bacterial wilt (Su et al., 2022a; Su et al., 2022b).

Fortunately, the biological control of tomato bacterial wilt has become a hot research topic recently. Plant growth-promoting rhizobacteria (PGPR) are highly regarded by researchers as beneficial soil bacteria that colonize the plant rhizosphere and its surrounding environment among a diverse range of soil microorganisms. PGPR facilitate plant growth through both direct and indirect mechanisms, and play a crucial role in managing soil-borne plant diseases (Zhu et al., 2023). The application of *Bacillus velezensis* GL3 demonstrated significant suppression of *R. solanacearum* and enhanced the growth of tomato seedlings in a pot experiment (Agarwal et al., 2020). The results from both pot and field experiments indicated the significant suppression of tomato bacterial wilt by *Bacillus methylotrophicus* DR-08 through the secretion of two antimicrobial metabolites (clostridium difficile and clostridium difficile oxide) (Im et al., 2020). Ling et al. (2020) demonstrated the potential of *Streptomyces* sp. NEAU-HV9, and its secreting actinomycin D as effective biocontrol and antimicrobial agents against tomato bacterial wilt. NEAU-HV9 exhibits potential as biocontrol and novel antimicrobial agents against tomato bacterial wilt. The causes of *R. solanacearum* include the reduction in the number of beneficial bacteria in plant roots, the decline in bacterial diversity, and the accumulation of soil-borne pathogens. Hence, increasing the bacteria with strong beneficial functions in soil is particularly important for the prevention and control of tomato bacterial wilt. The utilization of composite microbial agent has been demonstrated to be more efficacious in the management of plant diseases compared with single microorganism, as indicated by several studies (Lee et al., 2017; Santiago et al., 2017). Zhou et al. (2021) reported that the combined application of *T. virens* Tvien6 and *B. velezensis* X5 was found to exhibit superior efficacy in controlling tomato bacterial wilt compared with the individual bacterial agents. Li et al. (2020) found that the application of the composite microbial agent exhibited enhanced efficacy in managing various soil-borne diseases and promoting the growth of *Avena sativa, Medicago sativa, and Cucumis sativus* seedlings. Besides, the strains *B. velezensis* B63 and *Pseudomonas fluorescens* P142 were observed to significantly suppress the occurrence of tomato bacterial wilt (Elsayed et al., 2019). Previous studies have demonstrated the effective biocontrol capabilities of *Bacillus subtilis* SL-44 against *Colletotrichum gloeosporioides*, *Rhizoctonia solani*, *Fusarium oxysporum*, *Alternaria alternata*, and *Botrytis cinerea* and promoted plant growth (Chen et al., 2023a; Chen et al., 2023b). Additionally, *Enterobacter hormaechei* Rs-5 has been found to alleviate salt stress and promote plant growth. Currently, some composite microbial agents have been studied to control tomato bacterial wilt. However, the mode of action of how composite microbial agent control tomato bacterial wilt by colonizing root and regulating the root soil microbial community remains unknown.

Intriguingly, we have not investigated SL-44 and Rs-5 for tomato bacterial wilt control. Therefore, the objective of this study was to prepare Rs-5 and SL-44 composite microbial agent (EB) and then study the control of tomato bacterial wilt by systematically investigating the root chemotaxis, root surface colonization, and mutualistic beneficial interaction between the EB and the tomato seedlings. The primary focus of this study was: (1) to investigate the biological control of tomato bacterial wilt and the ecological behavior of the composite bacterial agent in *R. solanacearum*-infected rhizosphere soil; (2) to evaluate the effect of the EB on the soil microbial community; (3) to evaluate the effect of the EB on soil fertility after *R. solanacearum* infection. These results will provide supplementary data for the development and application of composite PGPR as biological control agent in tomato bacterial wilt.

## 2 Materials and methods

### 2.1 Strains and medium

The strains *E. hormaechei* Rs-5, *B. subtilis* SL-44 and *R. solanacearum* GMI1000 were used in this study. The strains *E. hormaechei* Rs-5 and *B. subtilis* SL-44 used were isolated from soil in Xinjiang, China. The strain *R. solanacearum* GMI1000 was provided by Fujian Agriculture and Forestry University. Therefore, we took the first letter “E” of *Enterobacter* and the first letter “B” of *Bacillus*, and combined them into “EB” to represent the composite microbial agent. The strain Rs-5 or SL-44 were cultured in Luria-Bertani (LB) liquid medium (10 g/L NaCl, 10 g/L peptone, 5 g/L yeast extract, and pH 7.2) with shaking at 30°C and 170 rpm for 36 h, resulting in a cell concentration reaching up to 10^10^ CFU/mL. The strain GMI1000 was cultured in the bacteria peptone glucose (BG) liquid medium (Chen et al., 2022) (10 g/L peptone, 1 g/L yeast extract, 1 g/L casamino acid, 10 g/L glucose, and pH 7.4), with shaking at 28°C and 200 rpm for 36 h, resulting in a cell concentration reaching up to 10^10^ CFU/mL. Preparation of EB: took equal volume of fermentation broth of the strain Rs-5 and SL-44 and mix well, centrifuged (8,000 g for 10 min at 4°C) and resuspend with sterile distilled water (the density of bacterial suspension was controlled at 10^10^ CFU/mL). The preparation of SL-44 bacterial suspension: the cultured bacterial solution was centrifuged (8,000 g for 10 min at 4°C) and then suspended with phosphate buffer solution (pH 7.3) to the OD of 0.5. The preparation of Rs-5 and EB bacterial suspension followed the same procedure as described above.

### 2.2 The inhibition ability of EB against GMI1000

The inhibition efficacy of EB against GMI1000 was determined by the plate confrontation method. The fermented strain GMI1000 solution (100 μL) was inoculated onto BG agar plates and spread evenly. Three sterilized round filter papers (*φ* 0.5 cm) were placed on each LB agar plate, followed by inoculation of 10 μL of the fermented strain Rs-5, SL-44, or EB solution onto the filter papers. The presence of a bacterial inhibition circle was observed following incubation at 30°C for 48 h. The presence of this inhibition circle indicates the ability of Rs-5, SL-44, or the EB inhibiting GMI1000 growth, and conversely, its absence suggests no inhibitory effect.

### 2.3 The colonization of EB in tomato root

The tomato seeds were purchased from the agricultural materials store in Lintong, Shaanxi, China and the variety is *Provence*. The tomato seeds were sterilized by immersion in 70% ethanol for 5 min and subsequently in 0.1% HgCl_2_ for 10 min, washed several times with sterile water. The soil substrate (vermiculite and perlite in a 3:2 mixture) was added to the plant tissue culture containers (800 mL) and autoclaved (121°C for 20 min). The tomato seedlings were grown in the substrate until they had three true leaves, and then the tomato seedlings were removed and washed in sterile water to remove substrate from the roots. The seedlings were placed in 50 mL centrifuge tubes containing 20 mL bacterial suspension of strains Rs-5, SL-44, and EB. The centrifuge tubes were incubated in a climatic chamber (14/10 h light (350 μmol m^−2^ s^−1^)/dark period, and a relative humidity of 50%–60%) for 48 h. The tomato seedlings were taken out and their roots were washed with sterile water. Following to that, approximately 1 cm of sections were excised from distinct regions of the tomato roots. At the end, the tomato roots were fixed in 2.5% glutaraldehyde solution and subjected to freeze-drying before observation using scanning electron microscopy (SEM) (Hitachi, Japan).

### 2.4 Response of EB to simulated tomato root exudates

The specific root exudates selected in this study encompassed glucose, mannitol, sucrose, fructose, leucine, glutamic acid, aspartic acid, alanine, tartaric acid, malic acid, citric acid, and fumaric acid since the predominant constituents of tomato root exudates are sugar, amino acids, and organic acid. The response of Rs-5, SL-44, and EB to simulated tomato root exudates was evaluated by swarm plate assay (Yuan et al., 2015). The different simulated tomato root exudates (10 mM) were added to sterilized semi-solid medium (0.2% agar), shaken well and poured into petri dishes to cool. The concentrated solution was prepared by centrifuging (8,000 g for 10 min at 4°C) 10 mL cultured bacterial solution with 1 mL of phosphate buffer (pH 7.3), and then 20 μL the concentrated cell solution was taken in the center of the semi-solid medium. The blank control consisted of a semi-solid medium devoid of root exudates. The chemotactic radius was measured following overnight incubation in the above medium. If the composite microbial agent has a chemotactic response to the root exudate, the microbial cells will diffuse outward from the center of the medium, forming a white circle. Then, a ruler is used to measure the radius of the circle, which is the chemotactic radius.

### 2.5 Pot experiment

**Figure 1:**
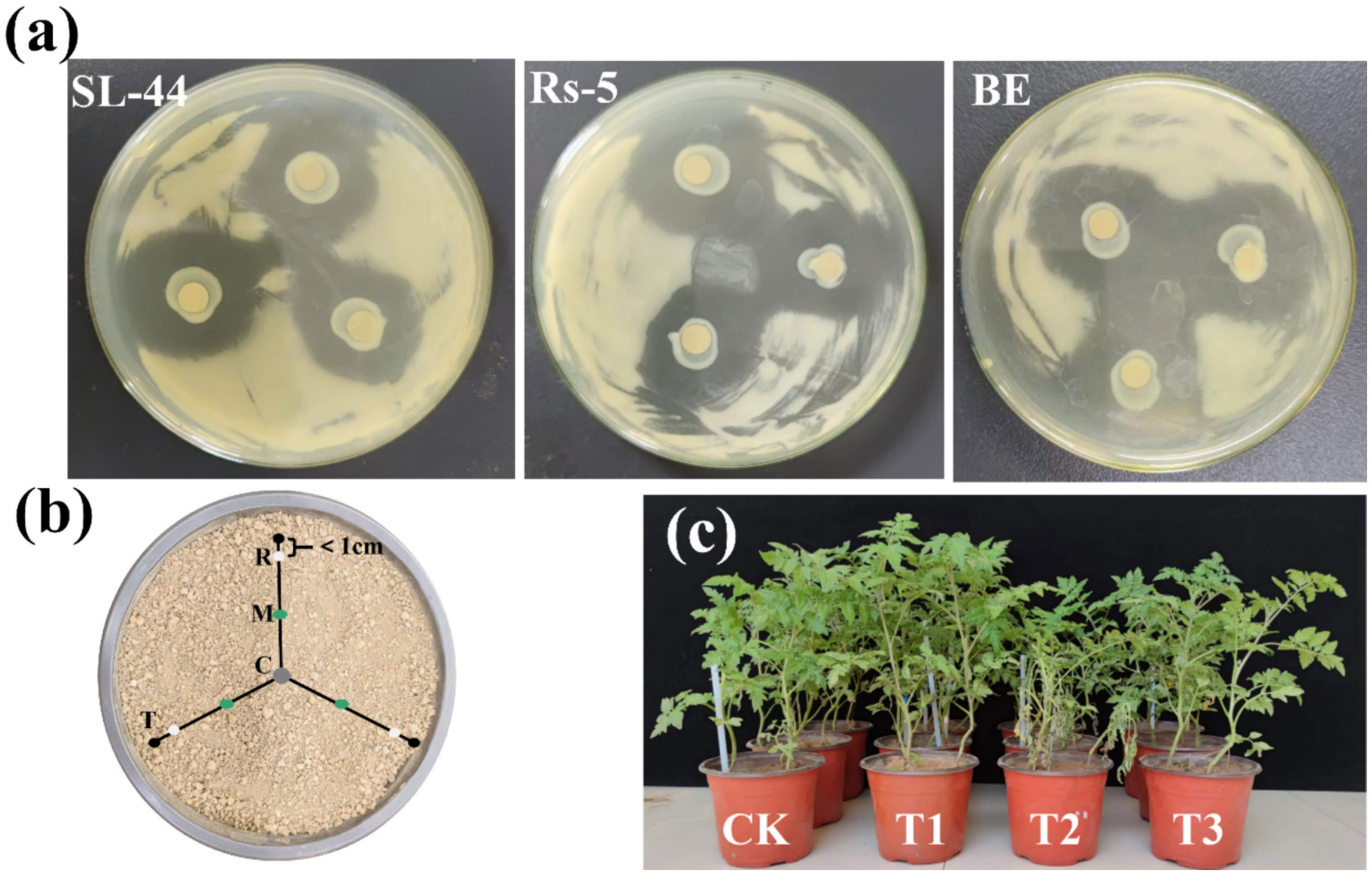
The inhibition abilities of the strain SL-44, Rs-5 and the composite microbial agent on GMI1000 **(a)**, Soil collection in pot **(b)** and the growth state of tomato seedlings transplanted for 30 days **(c)**. C, the inoculation site of the composite microbial agent; M, the middle of the tomato root and the composite microbial agent inoculation site; R, the tomato root; T, tomato seedlings.

The variety and the disinfection method of the seeds were the same as those mentioned above. The tomato seeds after disinfection were sown in nursery pots containing sterilized soil and transplanted when the tomato seedlings reached three leaves. The soil used for the pot experiments was from the campus of Xi’an Polytechnic University (34°21′N, 109°11′E), Shaanxi, China. The soil electrical conductivity (EC) was 480 μS·cm^−1^ and the pH was 7.90. The soil organic matter (SOM), soil alkali-hydrolytic nitrogen (SAN), soil available phosphorus (SAP), and soil available potassium (SAK) contents were 15.2 g·kg^−1^, 50.62 mg·g^−1^, 11.46 mg·kg^−1^ and 180 mg·kg^−1^, respectively. The pot experiments were divided into four groups as follows: CK, control without any treatment (no EB and pathogen GMI1000); T1, EB treatment; T2, pathogen GMI1000 treatment; and T3, EB and pathogen GMI1000 treatment. There were six pots (*φ* 23.5 cm × 19.5 cm) in each treatment and three tomato seedlings with consistent growth in each pot. The tomato seedlings transplanted to the T site in each pot (Figure 1b). The 3 kg of unsterilized soil in each pot. Before transplanting the tomato seedlings, the GMI1000 suspension was sprayed uniformly in the soil with a spray bottle, making the bacterial concentration about 10^7^ CFU g^−1^ soil for T2 and T3 treatment (5 mL GMI1000). The tomato seedlings were transplanted and inoculated with EB (the concentration about 10^8^ CFU g^−1^ soil) in the center of each pot. The seedlings were grown under room conditions with temperatures ranging from 26°C to 30°C, 14/10 h light (350 μmol m^−2^ s^−1^)/dark period, and a relative humidity of 50%–60%. The pot experiment ended after 30 days of tomato transplanted. The tomato plants were watered with deionized water by weighing method every 48 h during growth. The amount of water should be determined according to the weight of the soil and the seedling pot. The plant biomass (fresh weight, dry weight, plant height, root length, and stem diameter), physicochemical properties, and root soil nutrient content were measured at the end of the pot experiment.

Soil sample collection: Two sampling sites were set up to study the migration of the EB in the soil and its effect on the soil microbial community. The site M was located in the middle of the tomato root and the inoculation site of EB (Figure 1b), and the soil around the tomato root (site R). Soil at M and R was taken from three pots for each treatment at 15th and 30th day of the tomato transplanted. It was approximately 1 cm away from the root system of the tomato seedlings when taking the soil sample at the R site. It was approximately 7.5 cm away from the root system of the tomato seedlings when taking the soil sample at the M site. Three pieces of soil (9 g) at M and R were taken from each pot, and then were mixed well. The soil was stored at −80°C after removing impurities such as plant roots from the soil using a 2-mm sieve. The incidence of the tomato bacterial wilt was counted at 15th and 30th day of transplanting. The tomato symptoms were divided into 6 levels according to the proportion of leaf and stem wilt in the whole plant. *n* = 0, no symptoms; *n* = 1, 20% of the whole plant showed wilt symptoms; 2, 40% of the whole plant showed wilt symptoms; 3, 60% of the whole plant showed wilt symptoms; 4, 80% of the whole plant showed wilt symptoms; 5, 100% of the whole plant showed wilt symptoms. The disease index (DI) and relative control efficiency (CE) were calculated by Equations (1, 2).


(1) $$\begin{array}{c} DI=\left(100\sum \left(\begin{array}{l} \mathrm{disease level}\;n\times \mathrm{number of}\\ \mathrm{plants of corresponding level} \end{array}\right)\right)\\ /\left(\begin{array}{l} 5\times \mathrm{total number of plants of}\\ \mathrm{corresponding treatment} \end{array}\right) \end{array}$$

(2) $$\begin{array}{c} CE\;\left(\%\right)=\left(\mathrm{control disease index}-\mathrm{treatment disease index}\right)\\ \times 100/\mathrm{control disease index} \end{array}$$

### 2.6 The biomass and physicochemical properties of seedlings

The biomass of tomato seedlings included fresh weight, dry weight, plant height, root length and stem diameter. Fresh samples were washed with distilled water, then blotted dry with paper and their fresh weight was measured. The fresh samples were subjected to drying at 90°C until a constant weight was achieved to measure their dry weight. Plant height and root length were measured using a straightedge while Vernier calipers were employed for measuring the stem diameter of the seedlings.

The soluble protein was quantified using the Coomassie Brilliant Blue G-250 method and soluble sugars were determined by the sulfuric acid-phenol method (He et al., 2018). The absorbance of the corresponding indicator solutions was measured at wavelengths of 595 and 485 nm, respectively. Subsequently, the contents were calculated using the corresponding standard curves to determine the levels of soluble protein and soluble sugar in plants. Chlorophyll and carotenoid content were determined spectrophotometrically as described by He et al. (2019). After placing 0.2 g of plant leaves in a solution containing 10 mL of 96% ethanol, shaking well, and protecting from light overnight, the extracts’ absorbance was measured at wavelengths of 666, 649, and 470 nm.

Peroxidase (POD) was measured by the guaiacol method, superoxide dismutase (SOD) was measured by NBT photoreduction method, and catalase (CAT) was measured by UV absorption colorimetric method (Wu et al., 2019). The leaves of 0.2 g seedlings were washed and placed in a pre-chilled mortar (containing pre-chilled 0.05 M, pH 7.8 phosphate buffer) to grind into a homogenate, and the supernatant after centrifugation was the enzyme solution. Then the enzyme solution was mixed with its corresponding reaction solution, and the activities of POD, SOD and CAT were quantified at wavelengths of 470, 560, and 240 nm, respectively.

### 2.7 Root soil nutrient content

Soil organic matter (SOM) content was determined by potassium dichromate oxidation external heating method, soil available potassium (SAK) content was determined by acetic acid-flame photometer, soil available phosphorus (SAP) content was determined by the molybdenum blue method, and soil alkali-hydrolytic nitrogen (SAN) content was determined by alkali-hydrolytic diffusion method (Lu et al., 2020).

### 2.8 Absolute quantitative PCR

The absolute quantities of EB and GMI1000 in the soil at M or R sites were determined using Absolute Quantitative PCR (AQ-PCR), with three replicates per treatment. The primers Rs-5 (F) 5′-CACATCCGACTTGACAGACC-3′ and Rs-5 (R)5′-TTACCCGCAGAAGAAGCAC-3′ were used to detect Rs-5. The primers SL-44 (F)5′-GCGTAGAGATGTGGAGGAA-3′ and SL-44 (R) 5′-TAGGATTGTCAGAGGATGTCA-3′ were used to detect SL-44. The primers GMI1000 (F)5′-TAACCCAACATCTCACGACA-3′ and GMI1000 (R) 5′-ATGCCACTAACGAAGCAGA-3′ were used to detect GMI1000.

Soil DNA was extracted from 0.5 g of each sample using the Mag-Bind® Soil DNA Kit M5635 (Omega Bio-tek, United States), followed by standard product preparation. PCR cycling system: DNA template (1 μL), 10 μM Primer F (0.3 μL), 10 μM Primer R (0.3 μL), 2 × SYBR real-time PCR premixture (7.4 μL) and ultrapure water (7 μL). PCR cycling conditions: Pre-denaturation (95°C, 5 min), high-temperature denaturation (95°C, 15 s) and low-temperature annealing (60°C, 30 s), and the above stages were cycled 40 times.

### 2.9 High-throughput sequencing using NovaSeq PE250

Soil DNA samples were sequenced using the illumina NovaSeq PE250 platform from Shanghai Personalbio Technology Co., Ltd. The primers 515F (5′-GTGCCAGCMGCCGCGGTAA-3′) and 907R (5′-CCGTCAATTCCTTTGAGTTT-3′) were used to amplify the V4-V5 variable region of the bacterial 16S rRNA gene. The primers ITS1F (5-CTTGGTCATTTAGAGGAAGTAA-3′) and ITS2R (5′-GCTGCGTTCTTCATCGATGC-3′) were used to amplify the ITS1-ITS2 variable region of fungi. The raw data generated by Trimmomatic after sequencing was spliced and filtered by flash (flash Version 1.2.11) to obtain clean data to remove the interfering data. Then, Operational Taxonomic Units (OTUs) clustering and species annotation were conducted based on the valid data with a 97% similarity threshold. Additionally, Alpha Diversity and Beta Diversity analyses were performed to explore variations in community structure among different samples or groups.

### 2.10 Statistics analysis

Statistical analysis of the experimental data was performed using Origin 2021. One-way analysis of variance (ANOVA) in IBM SPSS Statistics 27 was used to evaluate the differences among the four treatments in the pot experiment at *p* < 0.05. OTUs clustering was performed using Uparse (Uparse v7.0). Alpha Diversity and Beta Diversity analyses were performed using Qiime (Version 1.9.1). R (Version 4.0.3, pheatmap package) was used to plot heat maps and R (Version 4.0.3, vcd package) was used to plot ternary heat maps. R (Version 4.0.3, Venn Diagram package) was used to plot Venn diagrams.

## 3 Results and discussion

### 3.1 The biocontrol of tomato bacterial wilt by EB

In this study, we investigated the antagonism interaction between the EB and *R. solanacearum* GMI1000, as well as the biocontrol efficacy of EB against tomato bacterial wilt by the plate confrontation method and pot experiment, respectively. The antagonistic experiment between the EB and GMI1000 exhibited a distinct inhibition zone (Figure 1a), indicating significant inhibitory effects of Rs-5, SL-44, and the EB on GMI1000. The pot experiment demonstrated that the tomato seedlings remained disease-free after the 15th day of transplantation (Supplementary Table S1), which may be related to the relatively long incubation period of bacterial wilt (usually 2–3 weeks) and the absence of mechanical damage (Li et al., 2021). *Ralstonia solanacearum* mainly invades through the wounds of the roots. In this study, no mechanical damage was caused to the tomato seedlings during the transplantation process. Therefore, the colonization rate of GMI1000 may be slowed down, resulting in the disease incidence of bacterial wilt of tomatoes in four treatments being 0 after the 15th day of transplantation. However, the incidence and DI of bacterial wilt in T2 treatment which is only *R. solanacearum* inoculation reached as high as 77.78% and 60, which indicated that *R. solanacearum* seriously affected the healthy growth of tomato seedlings after 30th days of transplantation. The incidence and DI of bacterial wilt in T3 treatment significantly decreased to 22.22% and 13.33%, following the application of EB, indicating that EB significantly inhibited the *R. solanacearum* growth. Furthermore, the CE of the EB against bacterial wilt reached 77.78%. Thus, both the antagonistic experiment and pot experiment of the EB and GMI1000 demonstrated the effective control of bacterial wilt by the EB.

### 3.2 The migration of EB to tomato root

**Figure 2:**
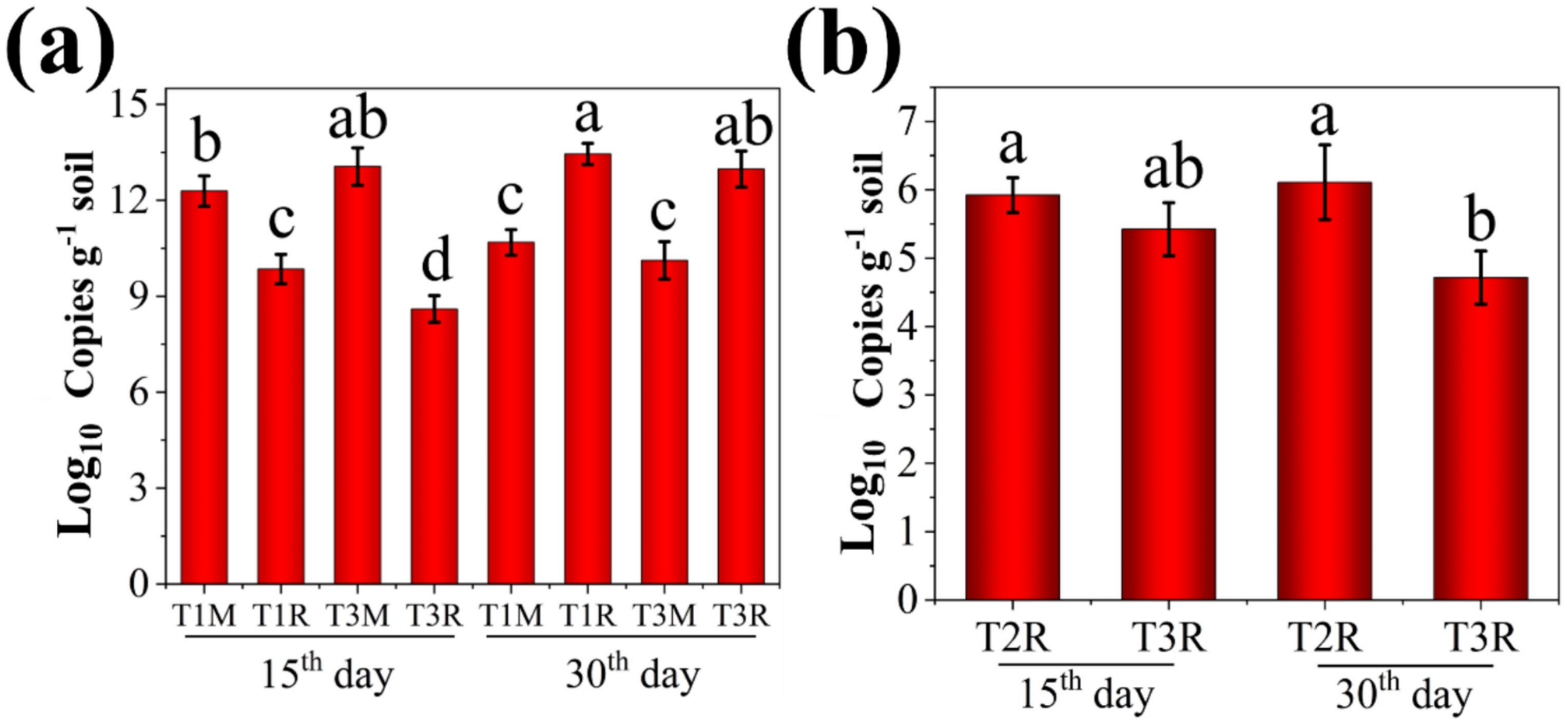
The quantities of composite microbial agent and GMI1000 in soil surrounding tomato. **(a)** The quantities of composite microbial agent at M or R sites; **(b)** The quantities of GMI1000 at R site. Lowercase letters indicate statistically significant differences between different groups. The same letters (such as a and a) indicate that there is no significant difference between the two groups, while different letters (such as a and b) indicate that there is a significant difference between the two groups (p < 0.05).

The migration ability of the EB toward tomato roots was assessed using AQ-PCR in this study, and the corresponding results are presented in Figure 2a. The amount of the EB in the M site for treatments, T1 and T3, was 12.29 and 13.06 (Log_10_ Copies g^−1^ soil), respectively, while in the R site it was 9.85 and 8.60 (Log_10_ Copies g^−1^ soil) on the 15th day after tomato transplanted. The amount of the EB in the M site for treatments, T1 and T3, was 10.68 and 10.12 (Log_10_ Copies g^−1^ soil), respectively, while in the R site, it was 13.45 and 12.98 (Log_10_ Copies g^−1^ soil) at the 30th day after tomato transplanted.

These results demonstrated that the amount of the EB at the R site in T1 and T3 treatments was lower than that of the M site at the 15th day after the tomato transplanted. Conversely, it was significantly increased at the 30th day after the tomato transplanted. AQ-PCR analysis suggested a gradual migration of the EB from its application site (C) toward tomato roots (R) over time. Bacteria have the chemotactic response to the root exudates of plants and can efficiently colonize the root surface, which can promote the migration of the composite microbial agent toward the tomato root in this study. Supplementary Figure S1 demonstrates that both strains Rs-5 and SL-44 exhibit a consistent change pattern at the M and R sites. However, it was observed a slight decrease in the R site in T3 treatment compared with that of T1 treatment at the 30th day after tomato transplanted, potentially attributed to the suppressive effect exerted by GMI1000 on the EB. In addition, the amount of GMI1000 in the rhizosphere soil was quantified for T2 and T3 treatments at the 15th and 30th day after tomato transplanted (Figure 2b). The amount of GMI1000 at the R site in T2 and T3 treatments was 5.92 and 5.42 (Log_10_ Copies g^−1^ soil) at the 15th day after tomato transplanted, respectively. The amount of GMI1000 at the R site in T2 and T3 treatments was 6.11 and 4.72 (Log_10_ Copies g^−1^ soil) at the 30th day after the tomato transplanted, respectively. The study found a progressive increase in the amount of GMI1000 at tomato root in T2 treatment over time. Conversely, the significant decrease (*p* < 0.05) was observed in the amount of GMI1000 at tomato root in the T3 treatment as time progressed. Overall, the increase of GMI1000 amount observed in the T2 treatment and the corresponding decrease in the T3 treatment with time provide compelling evidence that the EB can inhibit GMI1000 effectively. The GMI1000 may interfere with the colonization of EB through various pathways, such as competing for the carbon sources (amino acids and sugars) secreted by the roots, occupying the binding sites of the mucilage layer on the root surface, and secreting quorum sensing inhibitors to disrupt the formation of the biofilm of beneficial bacteria (Mendes et al., 2013). The migration of soil bacteria to the plant rhizosphere is the premise for its beneficial role in the plant rhizosphere (Liu et al., 2023). Similarly, the EB can migrate to the plant rhizosphere and decrease the amount of GMI1000, thus this is the premise of the EB to be effective in controlling tomato bacterial wilt in our study.

### 3.3 Chemotaxis toward tomato root exudates and tomato root colonization by EB

Plant root exudates not only provide nutrients for rhizosphere microorganisms but also serve as a driving force for microbial migration and colonization in the plant root system (Gu et al., 2020). The chemotactic movement of functional bacteria in microbial fertilizers toward the root system, induced by root exudates, represents the initial step in root colonization (Webb et al., 2017). The above study found the migration of the EB to the tomato rhizosphere, leading us to hypothesize that root exudates may serve as the chemoattractant for recruiting this EB. Therefore, we studied the chemotactic response of the EB toward various tomato root exudates (glucose, mannitol, sucrose, fructose, leucine, glutamic acid, aspartic acid, alanine, tartaric acid, malic acid, citric acid and fumaric acid) were studied using a swarm plate assay in this study.

The results demonstrated that strain SL-44 exhibited chemotaxis toward sucrose, glutamic acid, and aspartic acid, with respective chemotactic radii of 1.475, 1.975, and 2.325 cm (Table 1). Conversely, strain Rs-5 displayed chemotaxis toward leucine and glutamic acid, with corresponding chemotactic radii of 1.7 and 1.825 cm, respectively. Furthermore, the EB demonstrated chemotaxis toward sucrose, leucine, glutamic acid, and aspartic acid, with corresponding chemotactic radii of 1.125, 1.125, 2.05, and 1.225 cm, respectively. The amino acids (glutamic acid, leucine, and aspartic acid) exhibit a greater capacity to induce the movement of the EB compared to sugar (sucrose). Consequently, amino acids play a pivotal role in this migration process. The study by Feng et al. (2022) demonstrated that rhizosphere chemotaxis was primarily driven by a few key chemotactic factors rather than the cumulative effect of all chemotactic substances. Notably, strains Rs-5 and SL-44 exhibited significant chemotactic responses toward glutamate, with the largest observed chemotactic radius, thus glutamate was judged as the key chemotactic substance in this study. Root exudates encompass a wide array of signaling molecules and chemical inducers, which possess the ability to selectively recruit beneficial microbes to the root system for enrichment (Chen S. et al., 2023). Zhou et al. (2023) demonstrated that root exudates, particularly taxifolin-a flavonoid compound, can effectively induce the migration of *Bacillus* toward tomato roots. Wang et al. (2019) indicated that tomato root exudates (hexanoic acid and lactic acid) possess the ability to mitigate tomato bacterial wilt. Furthermore, it was observed that *Bacillus cereus* AR156 exhibited a chemotactic response toward fructose, lactic acid, sucrose and threonine present in tomato root exudates.

**Table 1:** The chemotactic response of composite microbial toward 12 root exudates of tomato.

Treatments		SL-44	Rs-5	Composite microbial agent
Root exudates				
Sugar	Glucose	-	-	-
	Mannitol	-	-	-
	Sucrose	1.475 + 0.25 (cm)	-	1.125 ± 0.395 (cm)
	Fructose	-	-	-
Amino acid	Leucine	-	1.7 ± 0.115 (cm)	1.125 ± 0.058 (cm)
	Glutamic acid	1.975 ± 0.171 (cm)	1.825 ± 0.236 (cm)	2.05 ± 0.05 (cm)
	Aspartic acid	2.325 ± 0.15 (cm)	-	1.225 ± 0.171 (cm)
	Alanine	-	-	-
Organic acid	Tartaric acid	-	-	-
	Malic acid	-	-	-
	Citric acid	-	-	-
	Fumaric acid	-	-	-

**Figure 3:**
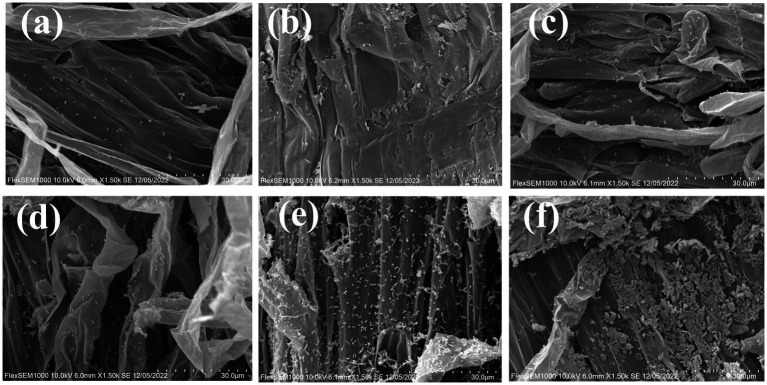
The colonization of composite microbial agent in tomato root surface. **(a–c)** The colonization of strain Rs-5, SL-44 and composite microbial agent in meristematic zone of tomato root; **(d–f)** The colonization of strain Rs-5, SL-44 and composite microbial agent in elongation zone of tomato root.

The above results have demonstrated the chemotactic effect of the EB on tomato root secretions, enabling its migration toward tomato roots in response to root exudates. Meanwhile, the colonization and biofilm formation of the EB on tomato roots are also crucial criteria for assessing its biocontrol efficacy. The SEM of the growth and elongation regions of tomato roots under different treatments were presented in Figure 3. The strain Rs-5, SL-44, and the EB exhibited a well clonization on the both the meristematic and elongation zones of tomato roots especially on the elongation zones. The biofilm was formed on the tomato root surface due to the large amount of colonization in the elongation zone of tomato root compared with Rs-5 and SL-44 (Figure 3f). Ye et al. (2020) also demonstrated the ability of *Corallococcus* sp. EGB to colonize and form biofilm in the elongated zone of *cucumber* roots, thereby effectively prevent cucumber wilt invade. Zhang and Shen (2022) proved that biofilm can resist adverse environmental factors, enabling them to grow and reproduce in unstable environment. Therefore, the efficient colonization of the EB in the elongation region enables effective control of tomato bacterial wilt in this study. Sampedro et al. (2020) demonstrated that *Halomonas anticariensis* FP35T performed beneficial function by root chemotaxis, efficient colonization and biofilm formation in the plant root.

### 3.4 The physicochemical properties and root soil nutrient content of tomato seedlings

**Figure 4:**
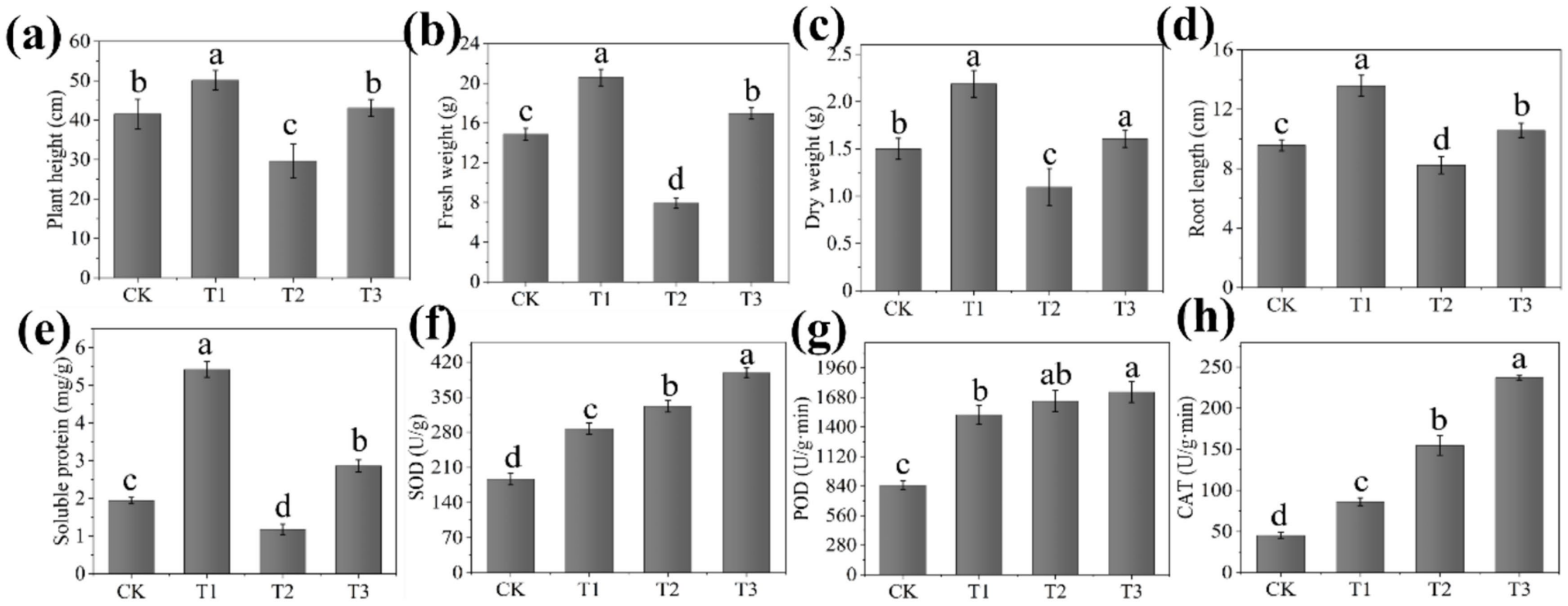
Effects of the composite microbial agent on biomass **(a–d)**, the soluble protein content **(e)**, and antioxidant enzyme activities **(f–h)** of seedlings (*n* = 6) Bars represent means standard error. Different lowercase letters indicate average of six replicates with significant differences at *p* < 0.05 among different treatments.

The assessment of plant biomass and physicochemical properties provides an intuitive way to validate the efficacy of the EB in managing tomato bacterial wilt. The growth state of tomato seedlings after 30 days of transplanting is depicted in Figure 1c, demonstrating the effective prevention and control of tomato bacterial wilt by the EB, as well as its growth promotion to healthy tomato seedlings. The plant height, fresh weight, dry weight, root length and stem diameter exhibited significant increases of 39.68%, 112.76%, 38.26%, 24.71%, and 29.27%, respectively, in the T3 treatment compared with the T2 treatment for the EB inoculation (Figures 4a–d; Supplementary Figure S2a). The plant height, fresh weight, dry weight, root length, and stem diameter exhibited significant increases of 19.38%, 35.04%, 37.24%, 27.55%, and 18.60%, respectively, in the T1 treatment compared to the CK treatment. The pot experiment demonstrated that the EB significantly enhanced the biomass of T3 treatment seedlings, which can be attributed to its effective suppression of GMI1000. The application of *B. subtilis* (SSR2I) also significantly enhanced the biomass of tomato seedlings infected with *R. solanacearum* (Jinal and Amaresan, 2020). Besides, the pro-growth properties of the EB such as phosphorus solubilization, nitrogen fixation, IAA, ACC deaminase and ferrophilin production may play a key role (Wu et al., 2019). Li et al. (2023) mentioned that the role of beneficial rhizosphere microbes in regulating root system growth and development, thereby facilitating plant growth.

In our study, the T3 treatment showed a significant increase in soluble protein (142.63%), soluble sugar (72.59%), chlorophyll (196.72%), and carotenoid contents (214.97%) compared with the T2 treatment (Figure 4e; Supplementary Figures S2b–d). Similarly, the T1 treatment showed a significant increase in soluble protein (178.91%), soluble sugar (51.90%), chlorophyll (23.35%), and carotenoid contents (22.17%) compared with the CK treatment. Soluble protein and sugar play a crucial role in regulating the osmotic regulation capacity of plant cells, thereby mitigating damage to the plant cell membrane under stressful conditions (Wang et al., 2022). In addition, the content of chlorophyll and carotenoid in plants are the important physiology index of plant (Zheng et al., 2016). However, it can be seen from the above results that the soluble protein, soluble sugar, chlorophyll and carotenoids content of plant in the T2 treatment are significantly reduced, which may be due to leaf atrophy and growth obstruction of tomato seedlings infected with *R. solanacearum*, resulting in a decline in their synthetic ability (Hahm et al., 2017). Fortunately, the corresponding contents of tomato seedlings were significantly increased after treatment with the EB. The SOD, POD, and CAT activities in the T3 treatment showed a significant increase of 19.99%, 5.22%, and 53.16% respectively, compared with the T2 treatment (Figures 4f–h). Moreover, the SOD, POD, and CAT activities in the T1 treatment showed a significant increase of 53.95%, 78.85%, and 90.21% respectively, compared with CK treatment. The antioxidant enzyme activity of tomato seedlings was significantly enhanced in the T1 treatment compared with the CK treatment, indicating that the EB itself could stimulate plants to increase the antioxidant enzyme activity. Additionally, in the T2 treatment, there was a significant increase antioxidant enzyme activity of tomato, which helped alleviate stress caused by *R. solanacearum*. The highest level of antioxidant enzyme activity was observed in tomato in T3 treatment, which could be attributed to the combined growth promoting effect of EB and alleviation of the biotic stress brought by *R. solanacearum*. The activities of SOD and POD were observed to increase following infection with *R. solanacearum*, while their activities were further elevated upon treatment with the composite microbial agent consisting of *Trichoderma virens* and *B. velezensis* (Zhou et al., 2021). Jinal and Amaresan (2020) demonstrated that *B. subtilis* SSR2I application significantly increased the activities of SOD and POD, thereby enhancing tomato’s resistance ability against to bacterial wilt. Besides, studies have revealed that the antioxidant enzyme system in the plant is activated in order to maintain the oxidative balance of the cells, as shown by the increased activity of SOD and the decomposition of superoxide anion into H_2_O_2_. At the same time, the POD and CAT activity are subsequently increased, and the generated H_2_O_2_ is further oxidized and decomposed into water, which eliminates the oxidative stress damage to the cells, thus promoting plant growth under stressful conditions (Qian et al., 2020; Shabaan et al., 2023). Therefore, the above findings align with the outcomes of our study.

The contents of soil organic matter (SOM), soil alkali-hydrolytic nitrogen (SAN), soil available phosphorus (SAP) and soil available potassium (SAK) in the T3 treatment root significantly increased by 11.58%, 14.71%, 27.28%, and 13.92% respectively, compared with the T2 treatment (Supplementary Figure S4). Similarly, the contents of SOM, SAP, and SAK in the T1 treatment showed significant increases of 16.68%, 10.05%, 34.04%, and 19.94% respectively, compared with the CK treatment. Soil nutrient contents serve as crucial indicators for assessing soil health and nutrient cycling, playing an integral role in evaluating soil fertility (Abdelkrim et al., 2020). The EB as the PGPR in this study, can symbiosis with plant roots, promote the growth and activity of soil microbes, and thus increase the soil organic matter content. Previous studies have found that the compound bacteria had the functions of nitrogen fixation, phosphorus dissolution and potassium dissolution, which will promote the increase of soil alkali-hydrolytic nitrogen, available phosphorus and available potassium in plant rhizosphere (Wu et al., 2019; Yue et al., 2007). Therefore, the increase of rhizosphere soil nutrient content in T3 treatment was due to the effective inhibition of EB on GMI1000.

In general, the pot experiment revealed a severe impact of bacterial wilt on the healthy growth of tomato seedlings, leading to a significant reduction in biomass, physicochemical properties, and rhizosphere soil nutrient content in plants subjected to T2 treatment. However, the addition of the EB effectively suppressed tomato bacterial wilt and significantly enhanced the physicochemical properties and rhizosphere soil nutrient content of tomato seedlings under T3 treatment.

### 3.5 Effects of EB on the microbial structure and diversity in tomato rhizosphere soil

The interactions between the inter-root soil microbiome and plants play a pivotal role in facilitating the robust growth and enhanced productivity of plants (Li et al., 2019). Herein, we investigated the impact of EB on the microbial community in the rhizosphere soil of tomato seedlings infected with *R. solanacearum*. We observed a gradual saturation of the number of OTUs in the four groups with increasing sequencing depth. The rarefaction curve for bacteria and fungi exhibited a gradual flattening trend (Supplementary Figure S4), indicating that the amount of sample sequencing data was appropriate and met the requirements of this study. At the 97% similarity level, bacteria and fungi detected a total of 4,119 and 1,810 OTUs, respectively, across all four treatments (Supplementary Figure S5). The Venn diagram revealed that the numbers of shared characteristic bacterial sequences in CK between T1, T2, and T3 treatments were 2,721, 2,581, and 2,681, respectively, (Supplementary Figure S5a). Similarly, the numbers of shared characteristic fungal sequences in CK between T1, T2, and T3 treatments were 538, 518, and 485, respectively, (Supplementary Figure S5b). The comparison between CK and T3 revealed a higher number of bacterial common feature sequences compared to that between CK and T2, indicating a relatively similar composition of bacterial OTUs between CK and T3. We speculated that the aforementioned phenomenon could be attributed to the stress-alleviating effects of the EB on the rhizosphere soil bacterial community in response to *R. solanacearum*. However, the common characteristic sequences of fungi between CK and T3 were relatively fewer, which can be attributed to the synergistic effect of the EB and *R. solanacearum* on the fungal community in rhizosphere soil.

**Figure 5:**
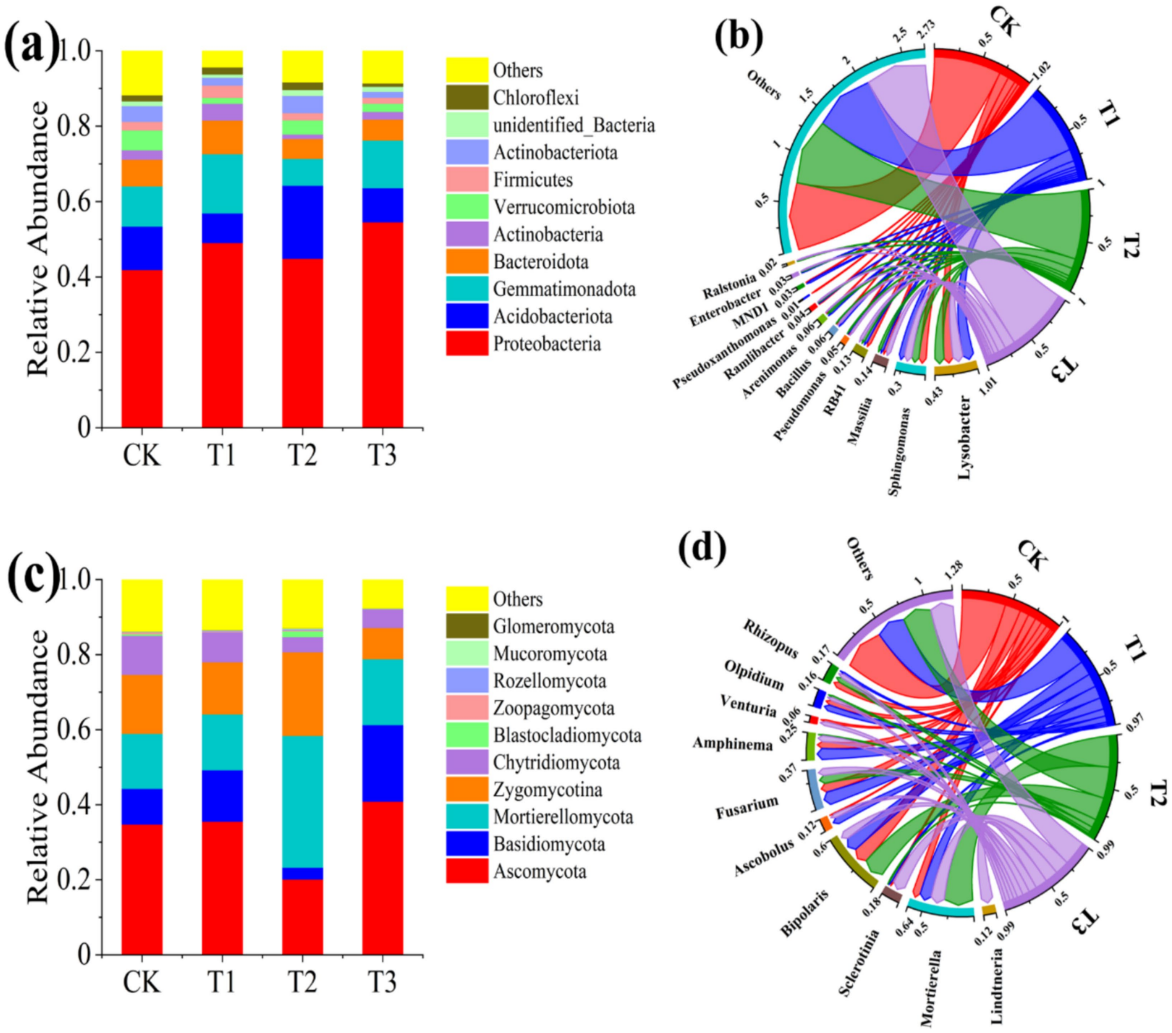
Changes in microbial community composition in in tomato rhizosphere soil. **(a)** Relative abundance of bacteria at the phylum level. **(b)** Relative abundance of bacteria at the genus level. **(c)** Relative abundance of fungi at the phylum level. **(d)** Relative abundance of fungi at the genus level.

The relative abundance of bacteria and fungi at phylum and genus levels in the rhizosphere soils of different treatments was depicted in Figure 5. In terms of bacterial community composition, 8 phyla of bacteria were detected with abundance more than 1%, namely *Proteobacteria*, *Acidobacteriota, Gemmatimonadota, Bacteroidota, Actinobacteria, Verrucomicrobiota, Firmicutes* and *Actinobacteriota* (Figure 5a). Among them, the relative abundance of *Proteobacteria* was highest in different treatments, with percentages of 41.94%, 49.06%, 44.88%, and 54.58% for CK, T1, T2, and T3, respectively. Similarly, *Proteobacteria* has been widely identified as the predominant bacterial group in diverse soil ecosystems (Zarraonaindia et al., 2015). Considering that both Rs-5 and GMI1000 belong to *Proteobacteria*, which no wonder that it resulted in the highest relative abundance of *Proteobacteria* in the T3 treatment. *Ralstonia solanacearum* exerted a significant impact on the relative abundance of bacteria at the phylum level. The abundance of *Acidobacteriota* significantly increased, whereas *Gemmatimonadota* and *Bacteroidota* exhibited a significant decrease in the T2 treatment compared with the CK treatment. However, the relative abundance of some phylum in the T3 treatment did not exhibit a statistically significant difference compared with that of CK treatment following administration of the EB. The 8 of the 10 most abundant bacterial genera found in soil samples were consistent across all four treatments, including *Lysobacter, Sphingomonas, Massilia, RB41, Pseudomonas, Bacillus, Arenimonas,* and *Ramlibacter*. Additionally, *Pseudoxanthomonas* was only detected in CK, *MND1* was present in both CK and T1, *Enterobacter* was present in T1, T2, and T3 while *Ralstonia* was present in T2 and T3 (Figure 5b). The detection of *Enterobacter* in the T1 and T3 treatments was expected; however, it was intriguing to find *Enterobacter* also present in the T2 treatment. We postulate that the *Enterobacter* present in T2 were recruited by the plant root exudates by system-induced resistance strategy to against *R. solanacearum*. Furthermore, both *Enterobacter* and *Ralstonia* were detected in the rhizosphere soil, providing additional evidence supporting our AQ-PCR findings for detecting Rs-5 and GMI1000 in plant rhizosphere soil. Hu et al. (2020) proved that the relative abundance of *Massilia* was higher in healthy soil compared with soil infected with *R. solanacearum*, which aligned with our findings. The relative abundance of *Pseudomonas* was significantly enhanced after EB inoculation when infected with *R. solanacearum*. The *Pseudomonas* sp. strain Y8 was also observed to exhibit significant efficacy in suppressing the growth of *R. solanacearum* (Li et al., 2021). Furthermore, our study revealed that the application of the EB led to a notable increase in the relative abundance of *Bacillus* within the tomato rhizosphere. Shen et al. (2021) also demonstrated the ability of *Streptomyces microflavus* G33 could enhance the relative abundance of *Bacillus* in the rhizosphere, thereby exerting inhibitory effects on tomato bacterial wilt. In general, the EB significantly enhanced the relative abundance of beneficial bacteria *Massilia, Pseudomonas, Bacillus* and *Enterobacter* in rhizosphere soil infected with *R. solanacearum*, thereby exerting a pivotal role in the management of bacterial wilt and promotion of healthy growth in tomato seedlings. Soil fungi are also integral components of the microbiome and play a pivotal role in maintaining the microecology of the rhizosphere, crucial for plant growth and development (Ali et al., 2020). The impact of *R. solanacearum* on the rhizosphere soil fungal community is evident in Figures 5c,d, particularly in terms of changes in relative abundance at the phylum and genus levels. The top 10 fungal phyla with higher relative abundance were A*scomycota, Basidiomycota, Mortierellomycota, Zygomycotina, Chytridiomycota, Blastocladiomycota, Zoopagomycota, Rozellomycota*, and *Glomeromycota*. *Ascomycota, Basidiomycota* and *Mortierellomycota* were the dominant phylum in the fungal community, which was consistent with other studies (Tortora et al., 2011; Shen et al., 2022). The short-term impact of the EB on the relative abundance of fungal community in the original soil sample was found to be statistically insignificant. The relative abundance of *Ascomycota, Basidiomycota* and *Chytridiomycota* was significantly decreased, while the relative abundance of *Mortierellomycota, Blastocladiomycota* and *Zygomycotina* was increased. On the contrary, the relative abundance of *Ascomycota* and *Basidiomycota*, and the relative abundance of *Mortierellomycota, Blastocladiomycota,* and *Zygomycotina* were significantly increased and reduced in tomato rhizosphere soil infected with *R. solanacearum* after application of the EB. The top 10 genera exhibiting high fungal relative abundance in the rhizosphere soil of each treatment were *Lindtneria, Mortierella, Sclerotinia, Bipolaris, Ascobolus, Fusarium, Amphinema, Venturia, Olpidium* and *Rhizopus*.

**Figure 6:**
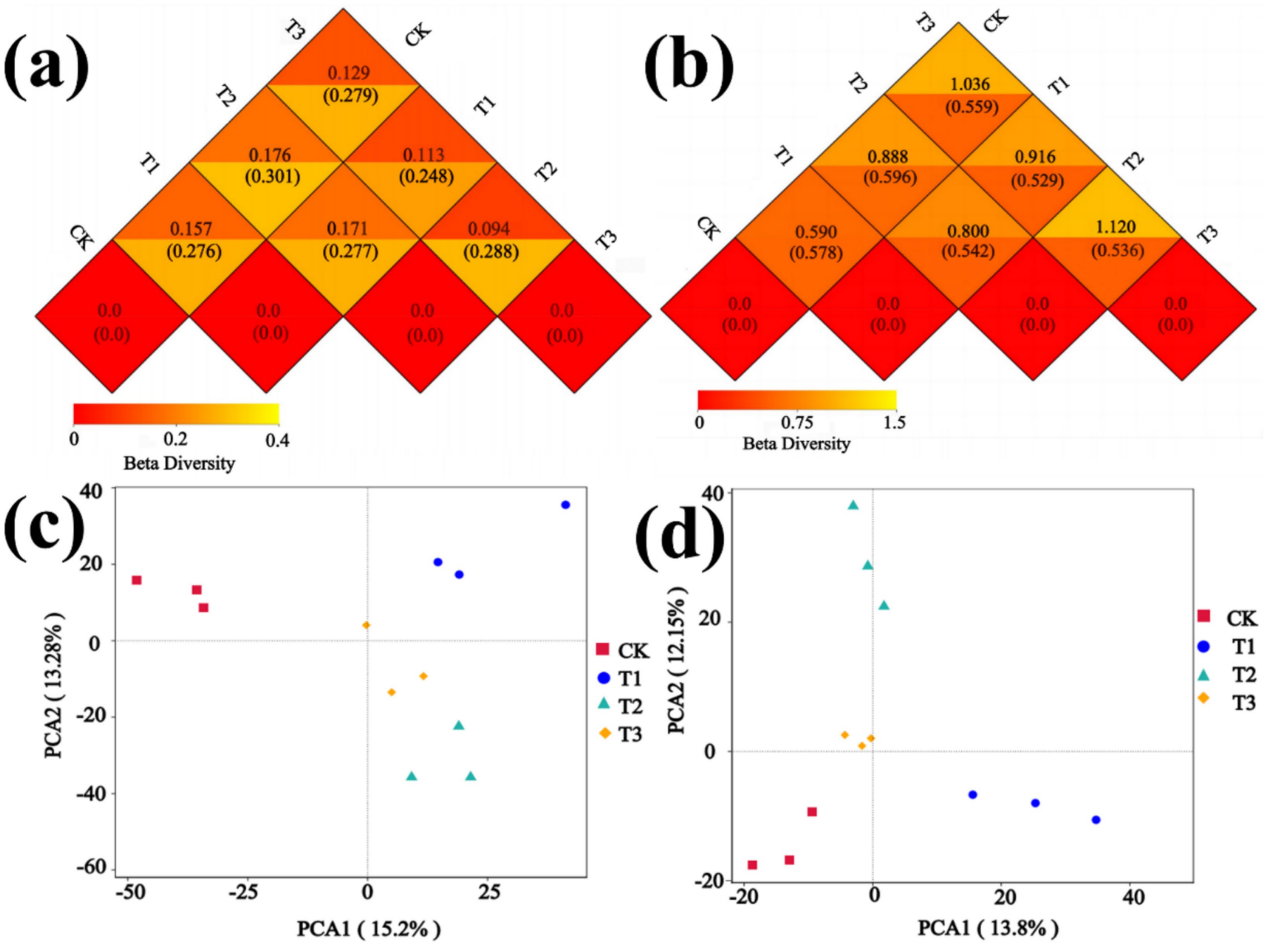
Beta diversity analysis of microbial community. **(a)** Distance Matrix Heatmap of bacteria. **(b)** Distance Matrix Heatmap of fungi. **(c)** PCA of bacteria. **(d)** PCA of fungi.

The diversity and dissimilarity of the rhizosphere soil microbial community were assessed using Alpha Diversity and Beta Diversity analyses. The bacterial Shannon index, Chao1 index, and Simpson index of the T1 treatment exhibited higher values compared to other treatments in Supplementary Figure S6a, while the T2 treatment displayed the lowest values for both bacterial Shannon index and Simpson index. The fungal Shannon index, Chao1 index, and Simpson index were found to be higher in the CK treatment compared to other treatments. Conversely, the fungal Shannon index, Chao1 index and Simpson index exhibited the lowest values in the T3 treatment (Supplementary Figure S6b). The fungal and bacterial Goods coverage index of the four treatments were approximately equal to 1. Bacterial and Fungal Beta Diversity were assessed using Weighted unifrac distance and Unweighted unifrac distance, which quantified the dissimilarity in species diversity between the two treatments. Additionally, Principal Component Analysis (PCA) was employed to evaluate the disparity in microbial community structure among the treatments. The results presented in Figure 6a demonstrate significant disparities in bacterial species diversity between the CK and T2, T1 and T2, while comparatively fewer differences were observed between T2 and T3. In Figure 6b, notable discrepancies in fungal species diversity were observed between CK and T3, T2, and T3. The bacterial community composition and structure exhibited variations among different treatments, as depicted in Figure 6c. The first and second axes represent the contribution values of the first and second principal components to the sample differences, explaining 15.2% and 13.28% of the overall variation in the bacterial community, respectively, with the total explanation of 28.48%. In addition, there were significant differences in the composition and structure of the fungal community were observed among different treatments (Figure 6d). The first and second axes represent the contribution values of the first and second principal components to the sample differences, explaining 13.8 and 12.15% of the overall variation in fungal community, respectively, with the total explanation of 25.95%.

## 4 Conclusion

The EB effectively inhibits tomato bacterial wilt, as evidenced by a significant reduction in the incidence and quantities of *R. solanacearum*, along with notable improvements in plant biomass, physicochemical properties, and rhizosphere soil nutrient contents. The EB was observed to exhibit migration toward the tomato root and colonization in the elongation zone, resulting in the formation of a film induced by root exudates, which serves as a prerequisite for its biocontrol efficacy. Furthermore, the EB can alter the microbial community structure in *R. solanacearum*-infected rhizosphere soil. In summary, the EB achieve their efficient control of tomato bacterial wilt goal through enriching in the rhizosphere, suppressing on *R. solanacearum*, and modulating the rhizosphere soil microbial community. Therefore, the utilization of composite microbial agent holds significant potential as the novel and efficient strategy for the control of tomato bacterial wilt.

## Data Availability

The original contributions presented in the study are included in the article/Supplementary material, further inquiries can be directed to the corresponding authors.

## Supplementary material

SUPPLEMENTARY FIGURE S1 The quantities of the stain SL-44 **(a)** and Rs-5 **(b)** at M or R sites.

SUPPLEMENTARY FIGURE S2 Effects of the composite microbial agent on stem diameter, soluble sugar, chlorophyll and carotenoid contents biomass of seedlings (*n* = 6).

SUPPLEMENTARY FIGURE S3 Effects of the composite microbial agent on the nutrient content of the root soil (*n* = 6).

SUPPLEMENTARY FIGURE S4 The rarefaction curve of bacteria **(a)** and fungi **(b)** in tomato rhizosphere soil.

SUPPLEMENTARY FIGURE S5 The 16S rRNA OTU Venn graph of bacteria **(a)** and fungi **(b)** in tomato rhizosphere soil.

SUPPLEMENTARY FIGURE S6 Alpha Diversity analysis of microbial community in tomato rhizosphere soil: **(a)** Shannon index, Chao1 index, Goods_coverage and Simpson index of bacteria community; **(b)** Shannon index, Chao1 index, Goods_coverage and Simpson index of fungi community.

SUPPLEMENTARY FIGURE S7 The mechanism of the composite microbial agent in the control of tomato bacterial wilt.
