# Supplementary material for: The composite microbial agent controls tomato bacterial wilt by colonizing the root surface and regulating the rhizosphere soil microbial community
Source: Front Microbiol. 2025 Apr 30;16:1559380. doi: 10.3389/fmicb.2025.1559380 (PMC12075239; doi:10.3389/fmicb.2025.1559380)
Supplement: Supplementary file 1 [file Table_1.docx]

Table.S1. Effect of composite microbial agent on the biocontrol of tomato bacterial wilt.

| Treatments | 15^th^ day | | | 30^th^ day | | |
| --- | --- | --- | --- | --- | --- | --- |
|  | Disease incidence(%) | DI | CE(%) | Disease incidence(%) | DI | CE(%) |
| CK | - | - | - | ﹣ | ﹣ | ﹣ |
| T1 | - | - | - | ﹣ | ﹣ | ﹣ |
| T2 | - | - | - | 77.78+19.24 | 60 | ﹣ |
| T3 | - | - | - | 22.22+19.24 | 13.33 | 77.78 |

Table.S2. The number of tomato seedlings infected with bacterial wilt at different levels.

| Treatments | | n | | | | | |
| --- | --- | --- | --- | --- | --- | --- | --- |
|  |  | 0 | 1 | 2 | 3 | 4 | 5 |
| CK | The number of tomato seedlings infected  with bacterial  wilt disease | 0 | 0 | 0 | 0 | 0 | 0 |
| T1 |  | 0 | 0 | 0 | 0 | 0 | 0 |
| T2 |  | 2 | 0 | 2 | 1 | 0 | 4 |
| T3 |  | 7 | 1 | 0 | 0 | 0 | 1 |
